# Why clinical trial outcomes fail to translate into benefits for patients

**DOI:** 10.1186/s13063-017-1870-2

**Published:** 2017-03-14

**Authors:** Carl Heneghan, Ben Goldacre, Kamal R. Mahtani

**Affiliations:** Centre for Evidence-Based Medicine, Nuffield Department of Primary Care Health Science, University of Oxford, Radcliffe Observatory Quarter, Woodstock Road, Oxford, OX2 6GG UK

**Keywords:** Clinical outcomes, Surrogate outcomes, Composite outcomes, Publication bias, Reporting bias, Core outcome sets

## Abstract

Clinical research should ultimately improve patient care. For this to be possible, trials must evaluate outcomes that genuinely reflect real-world settings and concerns. However, many trials continue to measure and report outcomes that fall short of this clear requirement. We highlight problems with trial outcomes that make evidence difficult or impossible to interpret and that undermine the translation of research into practice and policy. These complex issues include the use of surrogate, composite and subjective endpoints; a failure to take account of patients’ perspectives when designing research outcomes; publication and other outcome reporting biases, including the under-reporting of adverse events; the reporting of relative measures at the expense of more informative absolute outcomes; misleading reporting; multiplicity of outcomes; and a lack of core outcome sets. Trial outcomes can be developed with patients in mind, however, and can be reported completely, transparently and competently. Clinicians, patients, researchers and those who pay for health services are entitled to demand reliable evidence demonstrating whether interventions improve patient-relevant clinical outcomes.

## Background

Clinical trials are the most rigorous way of testing how novel treatments compare with existing treatments for a given outcome. Well-conducted clinical trials have the potential to make a significant impact on patient care and therefore should be designed and conducted to achieve this goal. One way to do this is to ensure that trial outcomes are relevant, appropriate and of importance to patients in real-world clinical settings. However, relatively few trials make a meaningful contribution to patient care, often as a result of the way that the trial outcomes are chosen, collected and reported. For example, authors of a recent analysis of cancer drugs approved by the U.S. Food and Drug Administration (FDA) reported a lack of clinically meaningful benefit in many post-marketing studies, owing to the use of surrogates, which undermines the ability of physicians and patients to make informed treatment decisions [1].

Such examples are concerning, given how critical trial outcomes are to clinical decision making. The World Health Organisation (WHO) recognises that ‘choosing the most important outcome is critical to producing a useful guideline’ [2]. A survey of 48 U.K. clinical trials units found that ‘choosing appropriate outcomes to measure’ as one of the top three priorities for methods research [3]. Yet, despite the importance of carefully selected trial outcomes to clinical practice, relatively little is understood about the components of outcomes that are critical to decision making.

**Fig. 1:**
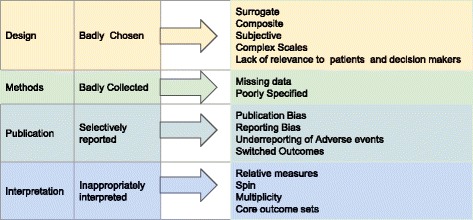
Why clinical trial outcomes fail to translate into benefits for patients

Most articles on trial outcomes focus on one or two aspects of their development or reporting. Assessing the extent to which outcomes are critical, however, requires a comprehensive understanding of all the shortcomings that can undermine their validity (Fig. 1). The problems we set out are complex, often coexist and can interact, contributing to a situation where clinical trial outcomes commonly fail to translate into clinical benefits for patients.

## Main text

### Badly chosen outcomes

#### Surrogate outcomes

Surrogate markers are often used to infer or predict a more direct patient-oriented outcome, such as death or functional capacity. Such outcomes are popular because they are often cheaper to measure and because changes may emerge faster than the real clinical outcome of interest. This can be a valid approach when the surrogate marker has a strong association with the real outcome of interest. For example, intra-ocular pressure in glaucoma and blood pressure in cardiovascular disease are well-established markers. However, for many surrogates, such as glycated haemoglobin, bone mineral density and prostate-specific antigen, there are considerable doubts about their correlation with disease [4]. Caution is therefore required in their interpretation [5]. Authors of an analysis of 626 randomised controlled trials (RCTs) reported that 17% of trials used a surrogate primary outcome, but only one-third discussed their validity [6]. Surrogates generally provide less direct relevant evidence than studies using patient-relevant outcomes [5, 7], and over-interpretation runs the risk of incorrect interpretations because changes may not reflect important changes in outcomes [8]. As an example, researchers in a well-conducted clinical trial of the diabetes drug rosiglitazone reported that it effectively lowered blood glucose (a surrogate) [9]; however, the drug was subsequently withdrawn in the European Union because of increased cardiovascular events, the patient-relevant outcome [10].

#### Composite outcomes

The use of combination measures is highly prevalent in, for example, cardiovascular research. However, their use can often lead to exaggerated estimates of treatment effects or render a trial report uninterpretable. Authors of an analysis of 242 cardiovascular RCTs, published in six high-impact medical journals, found that in 47% of the trials, researchers reported a composite outcome [11]. Authors of a further review of 40 trials, published in 2008, found that composites often had little justification for their choice [12], were inconsistently defined, and often the outcome combinations did not make clinical sense [13]. Individual outcomes within a composite can vary in the severity of their effects, which may be misleading when the most important outcomes, such as death, make relatively little contribution to the overall outcome measure [14]. Having more event data by using a composite does allow more precise outcome estimation. Interpretation, however, is particularly problematic when data are missing. Authors of an analysis of 51 rheumatoid arthritis RCTs reported >20% data was missing for the composite primary outcomes in 39% of the trials [15]. Missing data often requires imputation; however, the optimal method to address this remains unknown [15].

#### Subjective outcomes

Where an observer exercises judgment while assessing an event, or where the outcome is self-reported, the outcome is considered subjective [16]. In trials with such outcomes, effects are often exaggerated, particularly when methodological biases occur (i.e., when outcome assessors are not blinded) [17, 18]. In a systematic review of observer bias, non-blinded outcome assessors exaggerated ORs in RCTs by 36% compared with blinded assessors [19]. In addition, trials with inadequate or unclear sequence generation also biased estimates when outcomes were subjective [20]. Yet, despite these shortcomings, subjective outcomes are highly prevalent in trials as well as systematic reviews: In a study of 43 systematic reviews of drug interventions, researchers reported the primary outcome was objective in only 38% of the pooled analyses [21].

#### Complex scales

Combinations of symptoms and signs can be used to form outcome scales, which can also prove to be problematic. A review of 300 trials from the Cochrane Schizophrenia Group’s register revealed that trials were more likely to be positive when unpublished and unreliable and non-validated scales were used [22]. Furthermore, changes to the measurement scale used during the trial (a form of outcome switching) was one of the possible causes for the high number of results favouring new rheumatoid arthritis drugs [23]. Clinical trials require rating scales that are rigorous, but this is difficult to achieve [24]. Moreover, patients want to know the extent to which they are free of a symptom or a sign, more so than the mean change in a score.

#### Lack of relevance to patients and decision makers

Interpretation of changes in trial outcomes needs to go beyond a simple discussion of statistical significance to include clinical significance. Sometimes, however, such interpretation does not happen: In a review of 57 dementia drug trials, researchers found that less than half (46%) discussed the clinical significance of their results [17]. Furthermore, authors of a systematic assessment of the prevalence of patient-reported outcomes in cardiovascular trials published in the ten leading medical journals found that important outcomes for patients, such as death, were reported in only 23% of the 413 included trials. In 40% of the trials, patient-reported outcomes were judged to be of little added value, and 70% of the trials were missing crucial outcome data relevant to clinical decision making (mainly due to use of composite outcomes and under-reporting of adverse events) [25]. There has been some improvement over time in reporting of patient-relevant outcomes such as quality of life, but the situation remains dire: by 2010, only 16% of cardiovascular disease trials reported quality of life, a threefold increase from 1997. Use of surrogate, composite and subjective outcomes further undermines relevance to patients [26] and often accompanies problems with reporting and interpretation [25].

Studies often undermine decision making by failing to determine thresholds of practical importance to patient care. The smallest difference a patient, or the patient’s clinician, would be willing to accept to use a new intervention is the minimal clinically important difference (MCID). Crucially, clinicians and patients can assist in developing MCIDs; however, to date, such joint working is rare, and use of MCIDs has remained limited [27].

Problems are further compounded by the lack of consistency in the application of subjective outcomes across different interventions. Guidelines, for example, reject the use of antibiotics in sore throat [28] owing to their minimal effects on symptoms; yet, similar guidelines approve the use of antivirals because of their effects on symptoms [29], despite similar limited effects [30]. This contradiction occurs because decision makers, and particularly guideline developers, frequently lack understanding of the MCIDs required to change therapeutic decision making. Caution is also warranted, though, when it comes to assessing minimal effects: Authors of an analysis of 51 trials found small outcome effects were commonly reported and often eliminated by the presence of minimal bias [31]. Also, MCIDs may not necessarily reflect what patients consider to be important for decision making. Researchers in a study of patients with rheumatoid arthritis reported that the difference they considered really important was up to three to four times greater than MCIDs [32]. Moreover, inadequate duration of follow-up and trials that are stopped too early also contribute to a lack of reliable evidence for decision makers. For example, authors of systematic reviews of patients with mild depression have reported that only a handful of trials in primary care provide outcome data on the long-term effectiveness (beyond 12 weeks) of anti-depressant drug treatments [33]. Furthermore, results of simulation studies show that trials halted too early, with modest effects and few events, will result in large overestimates of the outcome effect [34].

### Badly collected outcomes

#### Missing data

Problems with missing data occur in almost all research: Its presence reduces study power and can easily lead to false conclusions. Authors of a systematic review of 235 RCTs found that 19% of the trials were no longer significant, based on assumptions that the losses to follow-up actually had the outcome of interest. This figure was 58% in a worst-case scenario, where all participants lost to follow-up in the intervention group and none in the control group had the event of interest [35]. The ‘5 and 20 rule’ (i.e., if >20% missing data, then the study is highly biased; if <5%, then low risk of bias) exists to aid understanding. However, interpretation of the outcomes is seriously problematic when the absolute effect size is less than the loss to follow-up. Despite the development of a number of different ways of handling missing data, the only real solution is to prevent it from happening in the first place [36].

#### Poorly specified outcomes

It is important to determine the exact definitions for trial outcomes because poorly specified outcomes can lead to confusion. As an example, in a Cochrane review on neuraminidase inhibitors for preventing and treating influenza, the diagnostic criteria for pneumonia could be either (1) laboratory-confirmed diagnosis (e.g., based on radiological evidence of infection); (2) clinical diagnosis by a doctor without laboratory confirmation; or (3) another type of diagnosis, such as self-report by the patient. Treatment effects for pneumonia were statistically different, depending on which diagnostic criteria were used. Furthermore, who actually assesses the outcome is important. Self-report measures are particularly prone to bias, owing to their subjectivity, but even the type of clinician assessing the outcome can affect the estimate: Stroke risk because of carotid endarterectomy differs depending on whether patients are assessed by a neurologist or a surgeon [37].

### Selectively reported outcomes

#### Publication bias

Problems with publication bias are well documented. Among cohort studies following registered or ethically approved trials, half go unpublished [38], and trials with positive outcomes are twice as likely to be published, and published faster, compared with trials with negative outcomes [39, 40]. The International Committee of Medical Journal Editors have stated the importance of trial registration to address the issue of publication bias [41]. Their policy requires ‘investigators to deposit information about trial design into an accepted clinical trials registry before the onset of patient enrolment’. Despite this initiative, publication bias remains a major issue contributing to translational failure. This led to the AllTrials campaign, which calls for all past and present clinical trials to be registered and their results reported [42].

#### Reporting bias

Outcome reporting bias occurs when a study has been published, but some of the outcomes measured and analysed have not been reported. Reporting bias is an under-recognised problem that significantly affects the validity of the outcome. Authors of a review of 283 Cochrane reviews found that more than half did not include data on the primary outcome [43]. One manifestation of reporting bias is the under-reporting of adverse events.

#### Under-reporting of adverse events

Interpreting the net benefits of treatments requires full outcome reporting of both the benefits and the harms in an unbiased manner. A review of recombinant human bone morphogenetic protein 2 used in spinal fusion, however, showed that data from publications substantially underestimated adverse events when compared with individual participant data or internal industry reports [44]. A further review of 11 studies comparing adverse events in published and unpublished documents reported that 43% to 100% (median 64%) of adverse events (including outcomes such as death or suicide) were missed when journal publications were solely relied on [45]. Researchers in multiple studies have found that journal publications under-report side effects and therefore exaggerate treatment benefits when compared with more complete information presented in clinical study reports [46], FDA reviews [47], ClinicalTrials.gov study reports [48] and reports obtained through litigation [49].

The aim of the Consolidated Standards of Reporting Trials (CONSORT), currently endorsed by 585 medical journals, is to improve reporting standards. However, despite CONSORT’s attempts, both publication and reporting bias remain a substantial problem. This impacts substantially the results of systematic reviews. Authors of an analysis of 322 systematic reviews found that 79% did not include the full data on the main harm outcome. This was due mainly to poor reporting in the included primary studies; in nearly two-thirds of the primary studies, outcome reporting bias was suspected [50]. The aim of updates to the Preferred Reporting Items for Systematic Reviews and Meta-Analyses (PRISMA) checklist for systematic reviews is to improve the current situation by ensuring that a minimal set of adverse events items is reported [51].

*Switched outcomes* is the failure to correctly report pre-specified outcomes, which remains highly prevalent and presents significant problems in interpreting results [52]. Authors of a systematic review of selective outcome reporting, including 27 analyses, found that the median proportion of trials with a discrepancy between the registered and published primary outcome was 31% [53]. Researchers in a recent study of 311 manuscripts submitted to *The BMJ* found that 23% of outcomes pre-specified in the protocol went unreported [54]. Furthermore, many trial authors and editors seem unaware of the ramifications of incorrect outcome reporting. The Centre for Evidence-Based Medicine Outcome Monitoring Project (COMPare) prospectively monitored all trials in five journals and submitted correction letters in real time on all misreported trials, but the majority of correction letters submitted were rejected by journal editors [55].

### Inappropriately interpreted outcomes

#### Relative measures

Relative measures can exaggerate findings of modest clinical benefit and can often be uninterpretable, such as if control event rates are not reported. Authors of a 2009 review of 344 journal articles reporting on health inequalities research found that, of the 40% of abstracts reporting an effect measure, 88% reported only the relative measure, 9% an absolute measure and just 2% reported both [56]. In contrast, 75% of all full-text articles reported relative effects, and only 7% reported both absolute and relative measures in the full text, despite reporting guidelines, such as CONSORT, recommending using both measures whenever possible [57].

#### Spin

Misleading reporting by presenting a study in a more positive way than the actual results reflect constitutes ‘spin’ [58]. Authors of an analysis of 72 trials with non-significant results reported it was a common phenomenon, with 40% of the trials containing some form of spin. Strategies included reporting on statistically significant results for within-group comparisons, secondary outcomes, or subgroup analyses and not the primary outcome, or focussing the reader on another study objective away from the statistically non-significant result [59]. Additionally, the results revealed the common occurrence of spin in the abstract, the most accessible and most read part of a trial report. In a study that randomised 300 clinicians to two versions of the same abstract (the original with spin and a rewritten version without spin), researchers found there was no difference in clinicians’ rating of the importance of the study or the need for a further trial [60]. Spin is also often found in systematic reviews; authors of an analysis found that spin was present in 28% of the 95 included reviews of psychological therapies [61]. A consensus process amongst members of the Cochrane Collaboration has identified 39 different types of spin, 13 of which were specific to systematic reviews. Of these, the three most serious were recommendations for practice not supported by findings in the conclusion, misleading titles and selective reporting [62].

#### Multiplicity

Appropriate attention has to be paid to the multiplicity of outcomes that are present in nearly all clinical trials. The higher the number of outcomes, the more chance there is of false-positive results and unsubstantiated claims of effectiveness [63]. The problem is compounded when trials have multiple time points, further increasing the number of outcomes. For licensing applications, secondary outcomes are considered insufficiently convincing to establish the main body of evidence and are intended to provide supporting evidence in relation to the primary outcome [63]. Furthermore, about half of all trials make further claims by undertaking subgroup analysis, but caution is warranted when interpreting their effects. An analysis of 207 studies found that 31% claimed a subgroup effect for the primary outcome; yet, such subgroups were often not pre-specified (a form of outcome switching) and frequently formed part of a large number of subgroup analyses [64]. At a minimum, triallists should perform a test of interaction, and journals should ensure it is done, to examine whether treatment effects actually differ amongst subpopulations [64], and decision makers should be very wary of high numbers of outcomes included in a trial report.

#### Core outcome sets

Core outcome sets could facilitate comparative effectiveness research and evidence synthesis. As an example, all of the top-cited Cochrane reviews in 2009 described problems with inconsistencies in their reported outcomes [65]. Standardised core outcome sets take account of patient preferences that should be measured and reported in all trials for a specific therapeutic area [65]. Since 1992, the Outcome Measures in Rheumatoid Arthritis Clinical Trials (OMERACT) collaboration has advocated the use of core outcome sets [66], and the Core Outcome Measures in Effectiveness Trials (COMET) Initiative collates relevant resources to facilitate core outcome development and user engagement [67, 68]. Consequently, their use is on the increase, and the Grading of Recommendations Assessment, Development and Evaluation (GRADE) working group recommend up to seven patient-important outcomes be listed in the ‘summary of findings’ tables in systematic reviews [69].

## Conclusions

The treatment choices of patients and clinicians should ideally be informed by evidence that interventions improve patient-relevant outcomes. Too often, medical research falls short of this modest ideal. However, there are ways forward. One of these is to ensure that trials are conceived and designed with greater input from end users, such as patients. The James Lind Alliance (JLA) brings together clinicians, patients and carers to identify areas of practice where uncertainties exist and to prioritise clinical research questions to answer them. The aim of such ‘priority setting partnerships’ (PSPs) is to develop research questions using measurable outcomes of direct relevance to patients. For example, a JLA PSP of dementia research generated a list of key measures, including quality of life, independence, management of behaviour and effect on progression of disease, as outcomes that were relevant to both persons with dementia and their carers [70].

However, identifying best practice is only the beginning of a wider process to change the culture of research. The ecosystem of evidence-based medicine is broad, including ethics committees, sponsors, regulators, triallists, reviewers and journal editors. All these stakeholders need to ensure that trial outcomes are developed with patients in mind, that unbiased methods are adhered to, and that results are reported in full and in line with those pre-specified at the trial outset. Until addressed, the problems of how outcomes are chosen, collected, reported and subsequently interpreted will continue to make a significant contribution to the reasons why clinical trial outcomes often fail to translate into clinical benefit for patients.

## Abbreviations

COMET: Core Outcome Measures in Effectiveness Trials
COMPare: Centre for Evidence-Based Medicine Outcome Monitoring Project
CONSORT: Consolidated Standards of Reporting Trials
FDA: Food and Drug Administration
GRADE: Grading of Recommendations Assessment, Development and Evaluation
JLA: James Lind Alliance
MCID: Minimal clinically important difference
OMERACT: Outcome Measures in Rheumatoid Arthritis Clinical Trials
PRISMA: Preferred Reporting Items for Systematic Reviews and Meta-Analyses
PSP: Priority setting partnership
RCT: Randomised controlled trial
WHO: World Health Organisation
